# Cardiorespiratory Temporal Causal Links and the Differences by Sport or Lack Thereof

**DOI:** 10.3389/fphys.2019.00045

**Published:** 2019-02-05

**Authors:** Marcel Młyńczak, Hubert Krysztofiak

**Affiliations:** ^1^Warsaw University of Technology, Faculty of Mechatronics, Institute of Metrology and Biomedical Engineering, Warsaw, Poland; ^2^Department of Applied Physiology, Mossakowski Medical Research Centre, Polish Academy of Sciences, Warsaw, Poland

**Keywords:** granger causality framework, athlete training adaptation biomarker, cardiac function, tidal volume, elite athletes

## Abstract

Fitness level, fatigue and adaptation are important factors for determining the optimal training schedule and predicting future performance. We think that adding analysis of the mutual relationships between cardiac and respiratory activity enables better athlete profiling and feedback for improving training. Therefore, the main objectives were (1) to apply several methods for temporal causality analysis to cardiorespiratory data; (2) to establish causal links between the signals; and (3) to determine how parameterized connections differed across various subgroups. One hundred elite athletes (31 female) and a control group of 20 healthy students (6 female) took part in the study. All were asked to follow a protocol comprising two 5-min sessions of free breathing - once supine, once standing. The data were collected using Pneumonitor 2. Respiratory-related curves were obtained through impedance pneumography, along with a single-lead ECG. Several signals (e.g., tidal volume, instantaneous respiratory rate, and instantaneous heart rate) were derived and stored as: (1) raw data down-sampled to 25*Hz*; (2) further down-sampled to 2.5*Hz*; and (3) beat-by-beat sequences. Granger causality frameworks (pairwise-conditional, spectral or extended), along with Time Series Models with Independent Noise (TiMINo), were studied. The connections enabling the best distinctions were found using recursive feature elimination with a random forest kernel. Temporal causal links are the most evident between tidal volume and instantaneous heart rate signals. Predictions of the “effect” variable were improved by adding preceding “cause” samples, by medians of 20.3% for supine and 14.2% for standing body positions. Parameterized causal link structures and directions distinguish athletes from non-athletes with 83.3% accuracy on average. They may also be used to supplement standard analysis and enable classification into groups exhibiting different static and dynamic components during performance. Physiological markers of training may be extended to include cardiorespiratory data, and causality analysis may improve the resolution of training profiling and the precision of outcome prediction.

## 1. Introduction

Comprehensive monitoring and testing of homeostatic processes, fitness level, fatigue, adaptation and recovery appears crucial for sports medicine practitioners to identify optimal training schedules, establish sufficient training loads and promote desirable progress and competitive performance (Meeusen et al., 2013; Halson, 2014; Coutts et al., 2017; Kellmann et al., 2018; Schneider et al., 2018).

The advance of medical devices and even wearable sensors makes it easy to quantify outputs. Training load indicators may come from the training set-up itself, from training equipment, accelerometers, etc. (Cardinale and Varley, 2017). The training schedule may be established objectively, and competitions produce a wealth of performance metrics.

The problem is in quantifying and analyzing the input information. Homeostasis is a capacious term. Fitness level and fatigue are largely subjective. Adaptation and recovery can be estimated, but usually only regarding a specific parameter. It appears there is no holistic framework (Heidari et al., 2018).

One of the commonly used methods in daily practice is cardiac monitoring (Buchheit, 2014; Schmitt et al., 2015; Bellenger et al., 2016; Duking et al., 2016; Giles et al., 2016; Plews et al., 2017). Average heart rate and many heart rate variability parameters have been proposed to describe the resting, exercise and recovery states of the heart, to assess the training load (Saboul et al., 2016), to evaluate high vagal activity (Nakamura et al., 2016), to predict performance (Triposkiadis et al., 2009), to test the heart activity changes induced by endurance and athletic activities (Berkoff et al., 2007; Vanderlei et al., 2008), and to analyze over-training syndrome (Dong, 2016) or training adaptation (Plews et al., 2013).

Still, there are many doubts about implementing heart activity parameters, due to various studies yielding discordant results, using different courses of analysis or even over-interpreting (Schneider et al., 2018). Therefore, separate heart activity data can be used only for a few aspects of sports medicine.

The concept of network physiology is widely accepted (Bartsch et al., 2015), as cardiac parameters may be influenced by many factors, e.g., environmental, anatomical, physiological, psychological, demographic, etc. (Sandercock et al., 2005; Fatisson et al., 2016). There is also no clear consensus as to which coefficients are best in training response evaluation (Sala et al., 2017).

One testable combination is that of breathing and heart activity. The relationship between heart rate and ventilation is well described, but still very complex. The effect of breathing phasing is usually apparent in resting ECG as sinus respiratory arrhythmia (Larsen et al., 2010; Shaffer et al., 2014; McCraty and Shaffer, 2015). The alternate cardiorespiratory coupling, in which heartbeats seem to coincide with specific respiratory phases due to increased sympathetic nervous activity and changes in arterial blood pressure, has also been tested (Penzel et al., 2016; Sobiech et al., 2017). On the other hand, the baroreflex seems to adjust neural responses and affect both heart and respiratory activity (Reyes del Paso et al., 2013).

Separate use of both signals would not reveal significant information nor improve study resolution. Several parameters, like RMSSD, might be uninfluenced by tidal volume pattern, for both spontaneous and controlled breathing (Saboul et al., 2013), still being under the effect of respiratory rate (Schipke et al., 1999). Therefore, many methods of mutual signals analysis have been considered. Assessed frameworks have included time-, frequency- or information-domain parameterization; temporal, phase or causal relations; etc. (Jamšek et al., 2004; Lopes et al., 2011; Riedl et al., 2014; Gasior et al., 2016; Javorka et al., 2016; Müller et al., 2016; Kuhnhold et al., 2017; Sobiech et al., 2017; Wejer et al., 2017).

In our previous work, we tried to add another domain of interest, time-independent causality (Młyńczak and Krysztofiak, 2018). Our discoveries suggested different paths for lying supine (from tidal volume, through heart activity variation and average heart activity, to respiratory timing) than for standing (from normalized respiratory activity variation to average heart activity).

In a traditional approach, such a graph of connections is treated as an input. We proposed a different context: from a graph, one can indicate which interventions (changes in training schedule) may be applied to expect specific results. This is related to the “bottom-up” strategy of thinking about designing the training based on the optimal parameter that describes and shows adaptation status, not the inverse (Młyńczak and Krysztofiak, 2018).

Going into greater detail, the next step is temporal causality analysis. Granger-based causality or transfer entropy are the two most important methods (Faes et al., 2013; Porta et al., 2017; Valenza et al., 2018). In general, regardless of the details in various formulations, they may cover different aspects of relations between two time series, except for linear Gaussian processes, when they can be considered equivalent (Porta and Faes, 2016). The novel frameworks also include the generalization of the basic concept, in which the effect of zero-lag can also be considered and evaluated. Causal inference related to Time Series using Restricted Structural Equation Models (TiMINo) has also been introduced (Peters et al., 2013).

As the methods mentioned above can produce some parameters and indices, we hypothesize that they can be used to improve athlete profiles and analyze trends during a training period.

Therefore, the main objectives were:

- to apply several methods for temporal causality analysis to cardiorespiratory data;
- to establish causal links between the collected cardiac and respiratory signals; and
- to determine how parameterized connections differed across groups depending on the static and dynamic activity component during performance, or control.

## 2. Materials and Methods

### 2.1. Subjects and Device

A group of 100 elite athletes practicing different sports (31 female; mostly overlapping with the previous paper (Młyńczak and Krysztofiak, 2018), the difference in 5 subjects is coming from the need to ensure the stationarity of the signal for sufficient temporal causality analysis) and 20 healthy students (treated as a control group; 6 female) took part in the study, carried out at the National Centre for Sports Medicine in Warsaw during the routine periodic health evaluation and medical monitoring program, 3–4 months before the 2016 Olympic Games in Rio de Janeiro.

The sport types are defined according to Mitchell et al. (2005), where numbers refer to the static component of heart activity expressed as % of its maximal voluntary contraction (MVC):

- Low (I-29 subjects);
- Medium (II-42); and
- High (III-29);

and letters to the dynamic component (e.g., % of *VO*_2_*max*) occurring during competition:

- Low (A-5 subjects);
- Medium (B-45); and
- High (C-50).

The demographic descriptive statistics of the athletes are summarized in Table 1.

**Table 1:** The demographic summary of the study group; the sports types are defined according to Mitchell et al. (2005) and the description in Materials and Methods.

**Group (sport type)**	**N**		**Height [cm]**				**Body mass [kg]**			
	**Female**	**Male**	**Min**	**Mean**	**SD**	**Max**	**Min**	**Mean**	**SD**	**Max**
Control	6	14	157.0	176.0	7.5	186.0	52.0	66.5	9.9	91.0
IIIA	0	5	168.0	176.8	9.2	189.0	68.0	80.8	10.2	95.0
IB	4	20	170.0	193.5	11.2	208.0	61.0	82.9	12.9	104.0
IIB	7	2	167.0	174.7	9.4	193.0	55.0	65.0	15.0	98.0
IIIB	4	8	158.0	174.3	12.0	197.0	53.0	79.8	32.6	151.0
IC	1	4	169.0	176.0	9.3	190.0	55.0	71.8	13.0	85.0
IIC	11	22	162.0	185.3	12.8	207.0	49.0	81.2	17.9	115.0
IIIC	4	8	171.0	179.5	6.4	189.0	63.0	75.4	6.3	88.0

The procedure was approved by the Ethics Committee of Warsaw Medical University (permission AKBE/74/17). All participants were informed about the general aim of the measurements, and each athlete had previously signed a general consent form for the routine medical monitoring (consisting of a statement of acceptance of the use of the results for scientific purposes). The students provided separate written consent.

The data were collected using our device, Pneumonitor 2 (Młyńczak et al., 2017). This is the academically-developed prototype, intended for conducting research and teaching. Respiratory-related curves were obtained through impedance pneumography, along with a single-lead ECG (lead 2). The impedance data were measured using the tetrapolar method, with the electrode configuration proposed by Seppa et al. (2013). Standard Holter-type, disposable ECG electrodes were used. Both signals were sampled at 250*Hz*. Task Force (1996) stated it is the smallest sufficient in terms of R peak finding and heart rate variability analysis and over-sampled from a respiratory perspective.

### 2.2. Protocol and Pre-processing

The measurements were performed in a diagnostic room designated for cardiological examinations. All participants were asked to follow a protocol comprising:

- attachment of the electrodes,
- 10-min stabilization phase,
- 5-min session of spontaneous breathing while lying supine, and
- 5-min session of spontaneous breathing while standing.

None knew the impact of breathing and position on the study outcomes. The protocol is inspired by the orthostatic maneuver; however, we did not take into account several sub-periods (adaptation, recovery, etc.), but rather performed the analysis for the entire supine and standing segments.

ECG signals were processed by non-linear detrending for baseline alignment. Consecutive R peaks in each signal were then found based on the Pan-Tompkins algorithm. On the respiratory side, raw IP signals were first pre-processed by smoothing with a 1*s* averaging window (Młyńczak and Cybulski, 2017). Then, inspiratory and expiratory phases were detected from the differentiated, flow-related signal. We did not transform impedance into the volume, instead assuming that impedance changes reproduce the tidal volume signal in terms of shape (linear fitting provides the best agreement between IP and the reference, pneumotachometry) (Młyńczak et al., 2015). Therefore, no calibration was performed.

Next, three kinds of dataset (to assess the reliability of several temporal causality methods when applied to cardiorespiratory data) were calculated for each participant in each body position:

1. tidal volume (TV) + instantaneous respiratory rate (iRR) + and instantaneous heart rate (iHR), all down-sampled to 25*Hz* (for the temporal causal analysis of signals);
2. the same data further down-sampled to 2.5*Hz* (for the spectral causal analysis of signals); and
3. lengths of consecutive RR intervals + TV-related impedance amplitude + iRR + breathing phase, the last three measured at the R peaks (for the causal analysis of beat-by-beat sequences; 1 is an arbitrary value standing for inspiratory phase, –1 for expiratory, and 0 for pause).

The iRR was calculated by estimating intervals between consecutive inspiratory onsets, then interpolating to the initial sampling frequency. In the same manner, the iHR was calculated by estimating intervals between successive R peaks, then interpolating to fit the number of samples for TV data.

The first down-sampling was intended to reduce computational complexity (deep embedding in terms of samples), the second - to analyze the proper subrange of frequencies. Both were accompanied by suitable low-pass filtering. All analyses were carried out in MATLAB.

The stationarity analyses were performed on the signals from the first dataset using augmented Dickey-Fuller Test (in R, using *tseries* package, Trapletti and Hornik, 2018). As the null hypothesis is that the time series has a unit root, we considered the data to be stationary when *p* < 0.05.

### 2.3. Causality Analysis

Four methods of causality analysis were studied:

- Granger causality;
- Spectral Granger causality;
- Extended Granger causality; and
- Time Series Models with Independent Noise (TiMINo).

First three techniques are based on the Granger concept of causality intended for time series. The main idea of this approach is that X can be treated as a “cause” of Y if taking previous X values along with Y ones enables preparing the model, which predicts the next Y values better than by only taking previous Y values. The efficiency of the prediction can be parameterized by the variance of the differences between predicted and actual Y values. So-called G-causality combines the variances of two models with or without including X, and can be calculated with the equation (1):

(1) $$\begin{array}{r} {GC}_{x\to y,p}=ln\left(\frac{Var\left(\hat{y}∥y,p\right)}{Var\left(\hat{y}∥y+x,p\right)}\right) \end{array}$$

where p is the model order, and the arrow presents tested direction.

It appears that the G-causality parameter has F-distribution and the statistical test can be proposed to assess the significance of the predictability improvement when using X along with Y. When *p*-value is lower than an arbitrarily adopted threshold at the level of 0.05, X can be considered to be a “Granger-cause” of Y (Granger, 1980; Barnett and Seth, 2014).

The first approach, pairwise-conditional one (because of multivariate data), was applied to the first dataset and based on the MVGC framework (Barnett and Seth, 2014). It uses VAR modeling, and the best model order is chosen based on the automatically-established Bayesian Information Criterion (BIC), as presented in the Supplementary Material S1.

The second approach, pairwise-conditional spectral analysis, was applied to the second dataset and also uses MVGC framework (Barnett and Seth, 2014). The possibility of Granger analysis in the frequency domain is based on the concept of cross-power spectral density decomposition and utilizing the generalized definition of G-causality. The process (and also the justification of the usage of F-statistics for a conditional case) is described in detail in Geweke (1982) and Barnett and Seth (2014).

The third approach, extended Granger causality framework, is built on the same concept; however, differently as in the original case, it also takes into account zero-lag, instantaneous effects. This assumption came from physiological analyses, when phenomena take place within the same cycle, for cardiorespiratory conditions mostly affected by the parasympathetic system. The implementation is presented in detail by Schiatti et al. (2015). We applied the method to the third dataset, with an arbitrarily-established maximum lag of 4 (as R peak is a trigger it usually covers from the middle to the full respiratory cycle).

The last approach, Time Series Models with Independent Noise (TiMINo), is based on generalized additive models (GAMs) as an extension of the Structural Equation Model framework to time series data (Peters et al., 2013). Not like it is in the Granger definition, which exploits the residual variance, TiMINo models require independent residual time series. Another aspect is the model class restriction to additive noise ones. As described by Peters et al. (2013) both lagged and instantaneous effects can be found and so-called unfaithful feedbacks between the time series may be also deduced. Peters et al. (2013) showed that when the data are causally insufficient or the proposed model is misspecified, this method will avoid incorrect answers. Additionally, this method is not built on the asymmetry of time direction but rather considers identifiability emerging from restricted structural equation models (Peters et al., 2013). Similarly, this approach was also applied to the third dataset, with a maximum lag of 4.

Then, we calculated the G-causality values with the first and third methods and determined the peak amplitude and frequency of spectral G-causality with the second method. All values were stored only when statistically significant. Granger causality methods were implemented in MATLAB; to ensure reproducibility, the code used to calculate G-causalities from a hypothetical dataset, along with accompanying *p*-values, is provided as Supplementary Material S1. The G-causality values, along with demographic and descriptive information about the subjects, are provided as Supplementary Material S2.

From the G-causality, one can calculate the prediction improvement in the case where the cause variable is assumed in the model, based on Equation (2):

(2) Prediction Improvement = 100·eG-causality-100 [%]

The fourth method, TiMINo, was used to view the problem in a completely different context. We established causal links for the entire athlete and control groups and separately for both body positions, according to the TiMINo results.

The quantitative data (from the first three methods and from each connection, except for breathing phases and tidal volume for the third approach, which are dependent) enabling the best distinction between groups and between different competitive levels of activity components were found using Recursive Feature Elimination (RFE) with a random forest algorithm used to produce a model and estimate performance (10-fold cross-validation was implemented). The exploratory accuracy and Cohen's Kappa (without dividing the data into training and testing subsets) were calculated.

TiMINo, exploratory statistical analysis, and RFE were performed in R (R Core Team, 2018). The relevant code is provided as Supplementary Material S3.

**Figure 1:**
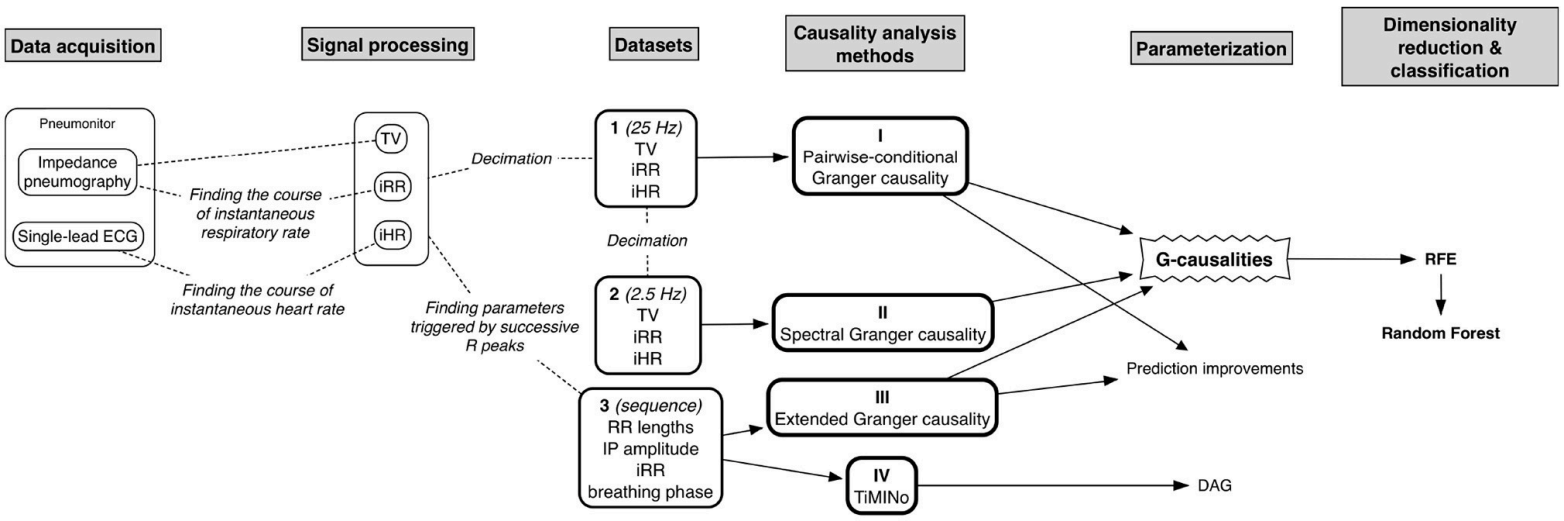
The diagram presenting the entire flow of the analysis.

The entire flow of the analysis is presented in Figure 1.

## 3. Results

### 3.1. General Findings

Granger causality analysis is originally intended for stationary data, therefore augmented Dickey-Fuller stationarity analysis was performed. It showed that:

- 2 TV-related signals during supine body position,
- 4 TV-related signals during standing body position,
- 1 iHR signal during supine body position, and
- 4 iHR signals during standing body position

can be labeled as non-stationary; however, it occurred jointly in no case.

**Figure 2:**
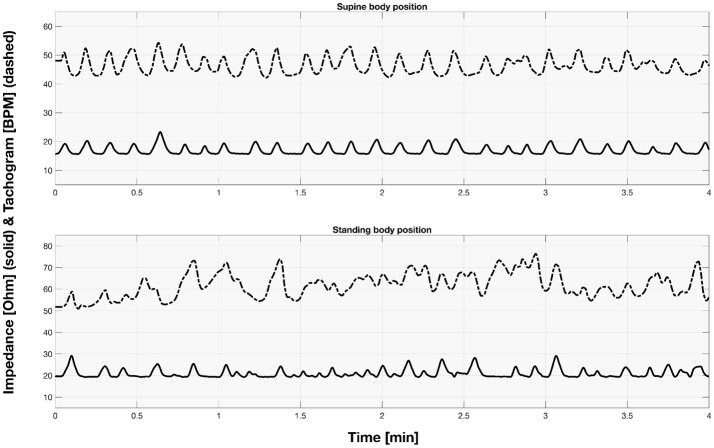
The sample part of impedance and tachogram signals acquired for the first participant for both supine and standing body position.

The sample signals acquired for both supine and standing body positions were presented in the Figure 2.

All the Granger-based methods revealed that iHR seems to cause TV. This can be due to the applied convention that inspiration causes the respiratory-related signal to increase, and expiration-to decrease. Therefore, at the instantaneous peak of heart rate during inspiration, the phase of iHR seems to precede that of TV. This is further debated in the Discussion section.

Apart from the spectral analysis, iRR appeared causally independent of iHR and TV, which may suggest that the heart activity is more related to the depth of breathing than to the rate.

### 3.2. Pairwise-Conditional Granger Causality

**Figure 3:**
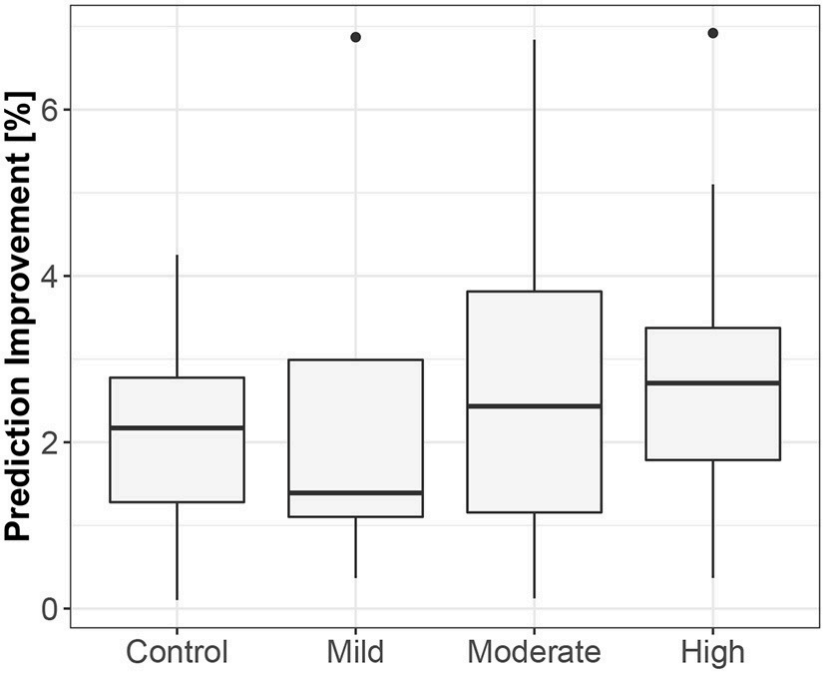
The exploratory box-plot presenting the G-causality values of the link from iHR to TV (in terms of % of prediction improvement of the models when taking causal information into account) for different competition levels of the dynamic component (and for the control group), while supine.

**Figure 4:**
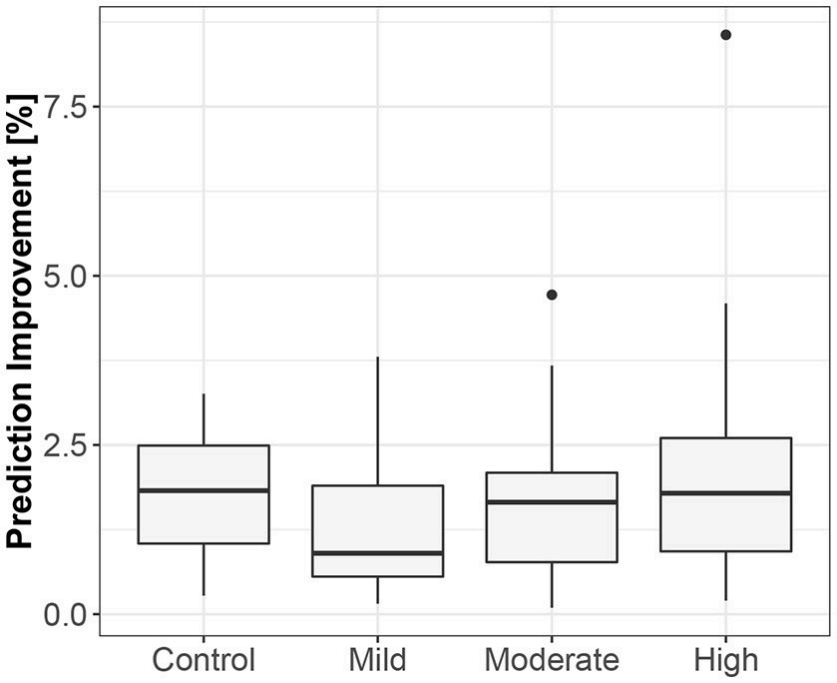
The exploratory box-plot presenting the G-causality values of the link from iHR to TV (in terms of % of prediction improvement of the models when taking causal information into account) for different competition levels of the static component (and for the control group), while standing.

The first method showed iHR causing TV changes, more for the supine body position (2.5% median prediction improvement; only 11 results out of 120 were statistically insignificant) than for standing (median of 1.7%; 20 insignificant results). The summary is stored in Table 2. For supine, the median G-causality for the athletes was greater than for the control group, but insignificantly so (*p* = 0.33 for the Wilcoxon rank test). The medians increased slightly, still insignificantly, for sports types with greater dynamic components (Figure 3; *p* = 0.81 for the Kruskal-Wallis test). For standing, the medians increased insignificantly for sports types with greater static components (Figure 4; *p* = 0.12 for Kruskal-Wallis). It is worth noting that the modest improvements are mainly caused by a relatively high sampling rate (from the perspective of the Granger causality framework). Inducing Granger causality appears quite insufficient when the sampling rate is so high compared to the physiological activity changes.

**Table 2:** The summary of prediction improvements (PI) for all considered directions for the first approach (raw signals sampled at 25*Hz*); 1. TV; 2. iRR; 3. iHR; NA, not assigned, if statistically insignificant (the more NAs, the more uncertain the link).

**Body position**	**Link**	**Mean PI**	**SD PI**	**Median PI**	**IQR PI**	**NA count**
Supine	1 → 2	0.24	0.12	0.19	0.19	69
	1 → 3	0.84	1.80	0.55	0.47	29
	2 → 3	0.58	0.43	0.41	0.60	30
	2 → 1	0.37	0.36	0.25	0.33	63
	3 → 1	**2.57**	**1.48**	**2.53**	**2.03**	**11**
	3 → 2	0.25	0.12	0.20	0.18	80
Standing	1 → 2	0.28	0.16	0.24	0.13	69
	1 → 3	0.47	0.40	0.37	0.38	46
	2 → 3	0.64	0.45	0.49	0.58	36
	2 → 1	0.45	0.33	0.34	0.31	58
	3 → 1	**1.70**	**1.28**	**1.67**	**1.51**	**20**
	3 → 2	0.21	0.10	0.17	0.10	99

*Bold values represent the most prominent and discussed link in the text*.

### 3.3. Pairwise-Conditional Spectral Granger Causality

The median peak frequency at which iHR influences TV was identified as 0.23*Hz* for lying supine (median G-causality equaled 0.82, vs. 0.27 for the opposite direction, only 3 results statistically insignificant) and 0.19*Hz* for standing (median G-causality of 0.68, vs. 0.14 for the opposite, 15 results insignificant). The second method also showed that iHR and TV seem to cause iRR at 0.04*Hz* on average, for both supine and standing. All directions are summarized in Table 3.

**Table 3:** The summary of medians and IQRs for peak G-causality and frequency for all considered directions for the second, spectral approach (raw signals sampled at 2.5*Hz*); 1. TV; 2. iRR; 3. iHR; G, G-causality; f, frequency in Hz; NA, not assigned, if statistically insignificant (the more NAs, the more uncertain the link).

**Body position**	**Link**	**Median G**	**IQR G**	**Median f**	**IQR f**	**NA count**
Supine	1 → 2	0.50	0.71	0.04	0.01	2
	1 → 3	0.27	0.37	0.10	0.06	2
	2 → 3	0.10	0.14	0.07	0.09	28
	2 → 1	0.04	0.04	0.21	0.12	63
	3 → 1	**0.82**	**0.65**	**0.23**	**0.12**	**3**
	3 → 2	0.23	0.49	0.04	0.01	7
Standing	1 → 2	0.45	0.65	0.04	0.02	4
	1 → 3	0.14	0.20	0.06	0.04	15
	2 → 3	0.14	0.20	0.07	0.03	15
	2 → 1	0.06	0.06	0.18	0.08	40
	3 → 1	**0.68**	**0.68**	**0.19**	**0.09**	**3**
	3 → 2	0.22	0.31	0.04	0.02	12

*Bold values represent the most prominent and discussed link in the text*.

### 3.4. Extended Granger Causality

**Figure 5:**
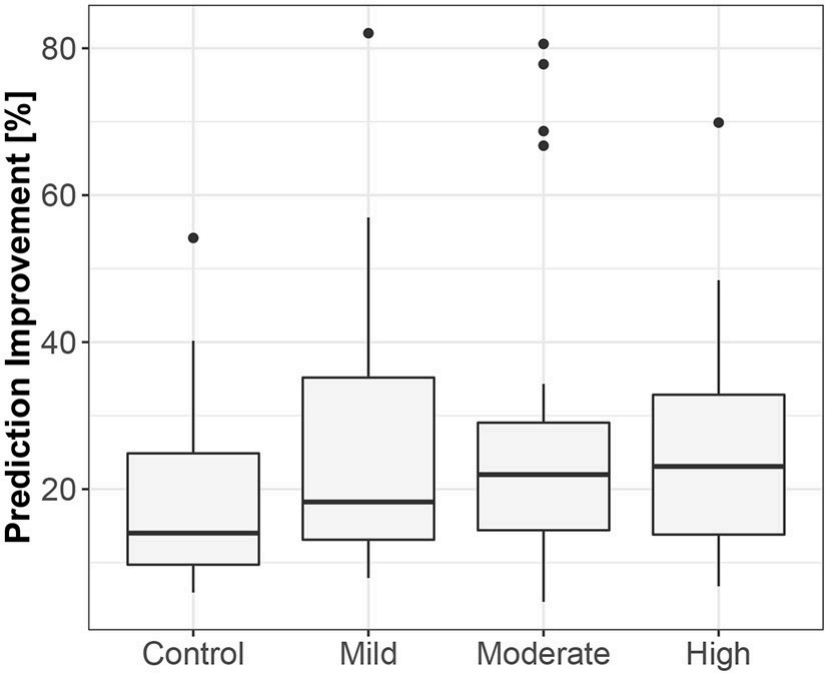
The exploratory box-plot presenting the G-causality values of the link from the lengths of successive RR intervals to the amplitudes of impedance at R peaks (in terms of % of prediction improvement of the models when taking causal information into account) for different competition levels of the static component (and for the control group), while supine.

**Figure 6:**
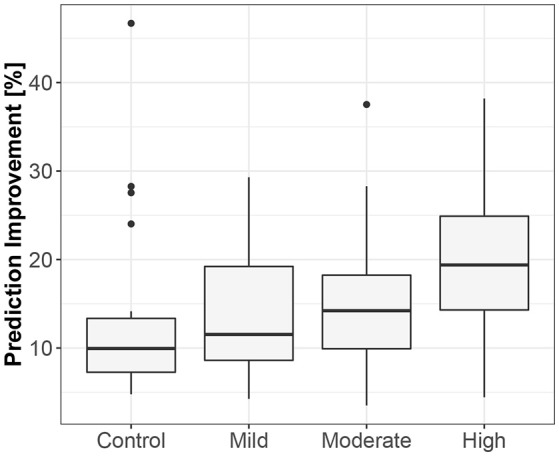
The exploratory box-plot presenting the G-causality values of the link from the lengths of successive RR intervals to the amplitudes of impedance at R peaks (in terms of % of prediction improvement of the models when taking causal information into account) for different competition levels of the static component (and for the control group), while standing.

The third method confirmed the direction to be from the lengths of successive RR intervals to the amplitudes of impedances at the R peaks. The median prediction improvement was 20.3% for supine (only 5 insignificant) and 14.2% for standing (also 5 insignificant). The summary appears in Table 4. For supine, the median G-causality value for the sports group was greater than for the control (*p* = 0.042 for Wilcoxon). Medians increased insignificantly for sports types with greater static components (Figure 5; *p* = 0.86 for Kruskal-Wallis). For standing, the median G-causality value for the sports group was greater than for the control (*p* = 0.042 for Wilcoxon). Medians increased significantly for sport types with greater static components (Figure 6; *p* = 0.028 for Kruskal-Wallis). Pair-wise, *post-hoc* Wilcoxon rank test indicated that significant differences appear between mild- and high-static-component groups (*p* = 0.048), and between moderate- and high-static-component groups (*p* = 0.048).

**Table 4:** The summary of prediction improvements (PI) for all considered directions for the third approach (beat-by-beat sequences); 1. Lengths of consecutive RR intervals; 2. TV-related impedance amplitude at R peaks; 3. iRR at R peaks; 4. Breathing phases at R peaks; NA, not assigned, if statistically insignificant (the more NAs, the more uncertain the link).

**Body position**	**Link**	**Mean PI**	**SD PI**	**Median PI**	**IQR PI**	**NA count**
Supine	1 → 2	**24.44**	**16.48**	**20.33**	**17.32**	**5**
	1 → 3	7.41	6.96	5.92	2.49	92
	1 → 4	16.71	14.09	11.34	14.08	29
	2 → 3	9.32	7.73	7.05	4.41	88
	2 → 4	69.15	39.76	56.99	54.43	2
	3 → 4	6.06	3.35	4.58	4.53	102
	2 → 1	19.42	25.74	12.60	11.81	40
	3 → 1	7.51	4.21	6.52	5.54	97
	4 → 1	11.58	8.95	8.16	7.04	66
	3 → 2	6.22	2.58	5.66	2.56	90
	4 → 2	34.50	21.88	33.56	26.08	9
	4 → 3	7.43	5.95	5.61	2.37	78
Standing	1 → 2	**15.37**	**8.40**	**14.18**	**12.15**	**5**
	1 → 3	4.34	2.81	3.49	1.89	105
	1 → 4	10.36	9.72	7.24	6.87	54
	2 → 3	6.63	3.28	4.90	3.85	89
	2 → 4	68.28	32.13	61.62	43.59	0
	3 → 4	8.05	8.69	4.84	3.88	103
	2 → 1	10.11	9.75	6.75	6.92	51
	3 → 1	10.91	12.58	5.72	9.61	97
	4 → 1	8.07	6.58	5.72	4.56	74
	3 → 2	5.85	3.58	4.18	3.82	100
	4 → 2	31.62	22.67	30.53	31.71	7
	4 → 3	5.73	3.22	4.40	3.52	93

*Very high numbers for the connections between 2 and 4 are ignored, due to their intrinsic relationship. Bold values represent the most prominent and discussed link in the text*.

The level of prediction improvement for the third approach, compared to the first, shows that Granger causality is more reliable for physiological beat-by-beat parameterizations than for raw signals sampled at 25*Hz*.

### 3.5. TiMINo

**Figure 7:**
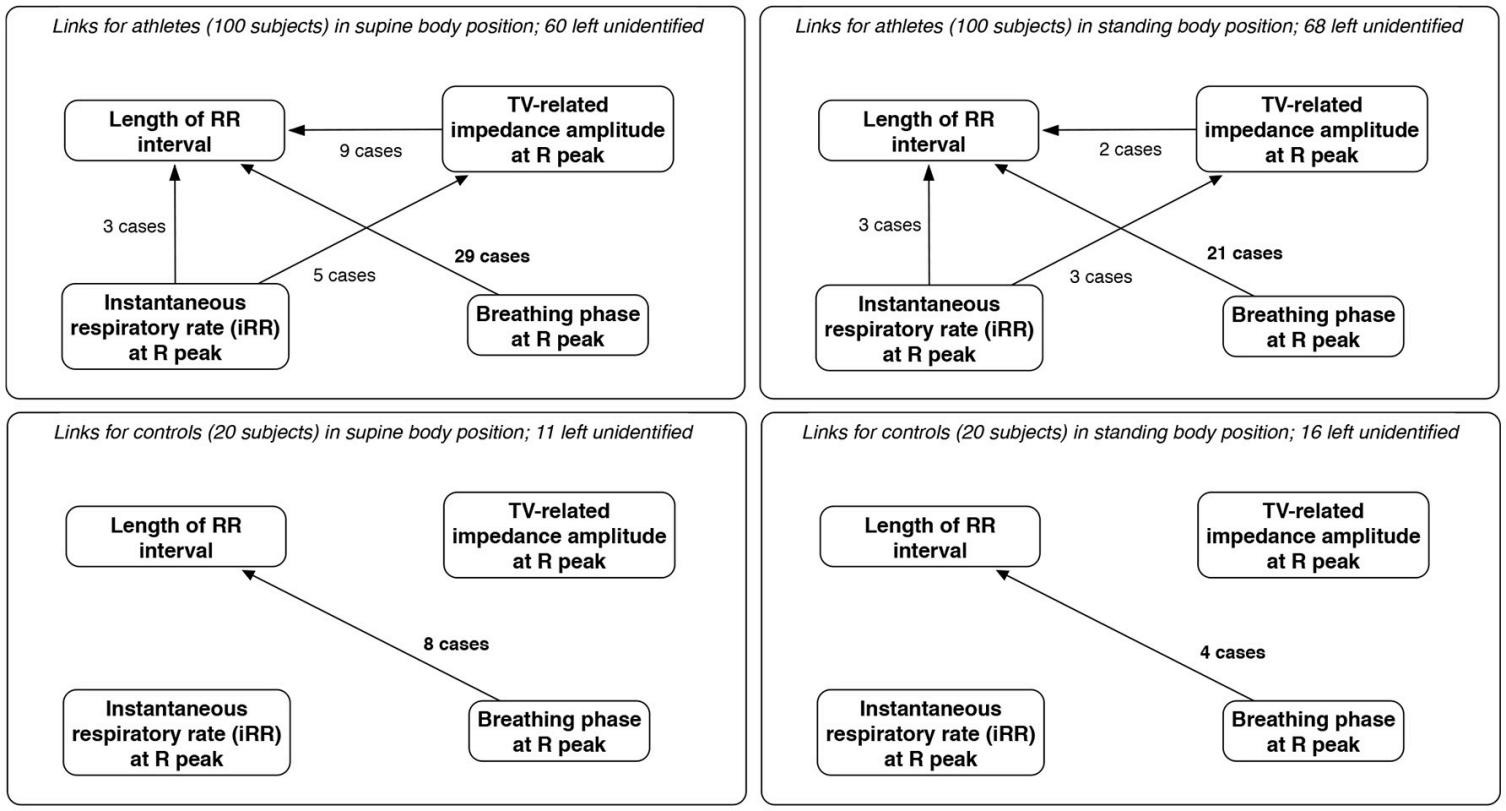
The links between parameters derived from beat-by-beat sequences for athletes and controls, for both supine and standing body positions, along with the number of cases in which each link was considered significant and the number where the method produced no results (unidentified output) due to independence of residuals or excessive model complexity relative to volume of available data. Only links significant in at least two cases are included; since breathing phases and volume are dependent, links between them are excluded, for clarity.

As expected for physiological data, many results of TiMINo analysis were left unidentified due to unfulfilled model assumptions (independence of residuals or model complexity too high for the amount of data available). The outputs suggested mostly that breathing phases cause the changes in lengths of consecutive RR intervals (29% for supine athletes, 21% for standing athletes, 40% for supine controls and 20% for standing controls). In this context, TiMINo seems to favor the respiratory sinus arrhythmia effect, no matter which convention of respiratory curve presentation is applied. Other connections are present, but only for a few cases, and only for athletes. The graphical summary is presented in Figure 7.

### 3.6. Distinguishing Sports or Lack Thereof

Recursive feature elimination suggested that 14 variables form the best set for distinguishing athletes and non-athletes (83.3±3.9% accuracy). The top five of these are:

- frequency at the peak of G-causality (2nd approach) from iHR to TV, while standing;
- G-causality value from the 1st approach, from TV to iRR, while standing;
- G-causality value from the 3rd approach, from lengths of consecutive RR intervals to breathing phases at R peaks, while standing;
- G-causality value from the 3rd approach, from lengths of consecutive RR intervals to TV-related impedance amplitudes at R peaks, while standing; and
- G-causality value from the 3rd approach, from lengths of consecutive RR intervals to TV-related impedance amplitudes at R peaks, while supine.

Another set was identified as the best for differentiating between moderate and high dynamic components during competition (we neglected low-dynamic-component-participants as there were only 5 subjects in this group (63.1±14.6% accuracy):

- frequency at the peak of G-causality (2nd approach) from iRR to iHR, while standing;
- G-causality value from the 3rd approach, from iRR at R peaks to breathing phases at R peaks, while supine;
- G-causality value from the 3rd approach, from iRR at R peaks to the breathing phase at R peaks, while standing;
- G-causality value from the 3rd approach, from TV-related impedance amplitude at R peaks to lengths of consecutive RR intervals, while standing;
- G-causality value from the 1st approach, from iRR to iHR, while standing; and
- G-causality value from the 1st approach, from iHR to TV, while supine.

Finally, a set of 30 variables appears best for analysis of various levels of the static component (51.8±12.3% accuracy), of which the top five are:

- G-causality value from the 3rd approach, from lengths of consecutive RR intervals to breathing phases at R peaks, while supine;
- G-causality value from the 3rd approach, from breathing phases at R peaks to iRR at R peaks, while supine;
- peak amplitude of G-causality (2nd approach) from iHR to iRR, while standing;
- peak amplitude of G-causality (2nd approach) from iRR to iHR, while standing; and
- frequency at the peak of G-causality (2nd approach) from iRR to TV, while supine.

## 4. Discussion

The main finding of our analysis is that the instantaneous heart rate (iHR) signal is causally related to the tidal volume (TV) signal. The Granger methods showed that iHR caused TV changes, in both time and spectral domains and for both raw signals and beat-by-beat sequences.

**Figure 8:**
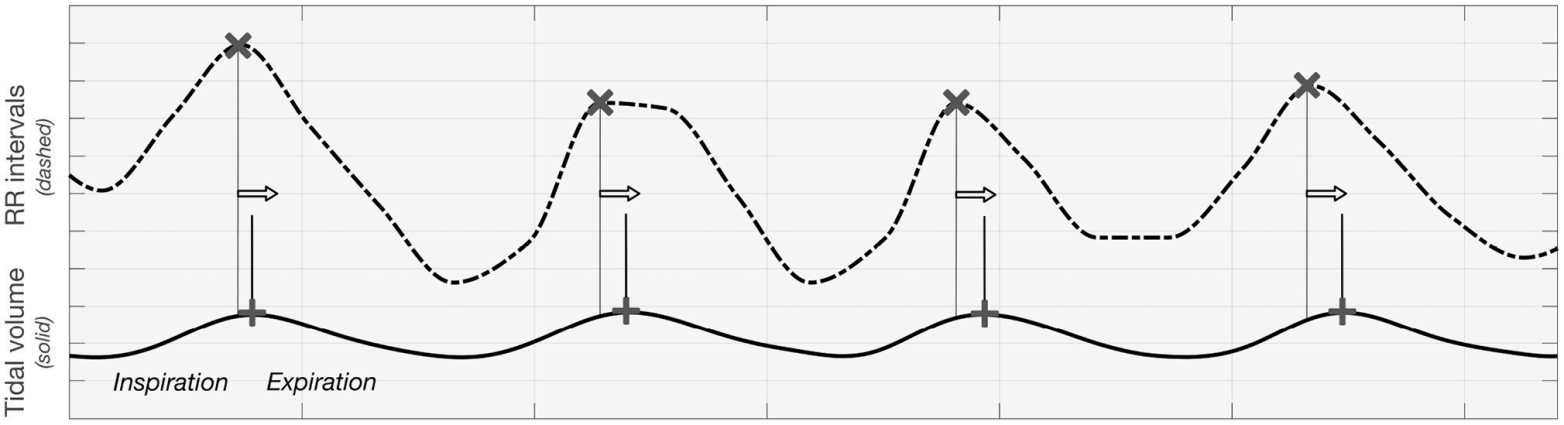
The visualization presenting the order of maxima when analyzing tidal volume and RR intervals curve.

As it is incoherent with the respiratory sinus arrhythmia effect (which is expected to have the largest impact, particularly during static supine; respiratory centers modulate the frequency of the heart through the vagal sinus node intervention (Eckberg, 2009) and the observations being the basis of the clinical autonomic screening tests (CARTs), we hypothesize that this is mainly due to the definition of the Granger causality-the cause should be before its effect. Relatively similar signal shape for RR intervals seems to appear before tidal volume, as the heart rate peak occurs during inspiration. In other words, the phase of iHR seems to precede that of TV when inspiration is presented as an increase of the signal's value. A visualization of this remark is presented in Figure 8.

Another method, Time Series Models with Independent Noise (TiMINo), suggested differently that breathing phases recorded at consecutive R peaks cause the lengths of RR intervals. As from the definition it is not based on the asymmetry of time, the RSA phenomenon is emphasized and confirmed (Shaffer et al., 2014; McCraty and Shaffer, 2015). An opposite effect, described by Sobiech et al. (2017), in which R peaks occur at strict intervals before the inspiratory onset, requires different mathematical approaches and different data preparation process.

The concept of analyzing both signals and sequences of parameters triggered by the heart activity with Granger methods is considered as a “test” of information capacity that can be extracted. The first approach is therefore inspired by recurrent, or even convolutional, deep learning methods, in which raw time series can be used as an input for the analysis. The results showed, however, that for signal-related analysis high computational complexity may distort the final inference. This is also most likely due to the redundancy that complicates the VAR modeling. Apart from that, the spectral analysis seems more proper when the intervals between the samples are equal.

We supposed that said relationships can be quantitatively parameterized to add extended data for sports training scheduling and monitoring. Koenig and Thayer (2016) found substantial differences between sexes regarding autonomic control of the heart. So why not explore the possible differences across sports, the levels of specific components during competition, static and dynamic conditions, body positions, etc.?

Therefore, we believe causality analysis may become a valuable and practical tool for trainers and physicians, as a method that not only finds or confirms causal connections and their directions, but can also parameterize them. As Schneider et al. (2018) wrote, training context is key. In our opinion, there are two steps to unlocking its promises. The first is to supplement the standard heart activity parameters in order to assess trends and competitive performance. Greenham et al. (2018) in their meta-analysis summarized that many biological and biochemical biomarkers varied with training intensity but not with performance. Several, like neutrophils, glutamine, urea and the testosterone/cortisol ratio, may be used to track performance. However, such approaches need not be individual. Cardiorespiratory data and analysis could be an objective addition, even more robust when combined with causality inference (it should not be used independently when the reported accuracies remain far from sufficient). This should be studied further.

The second is to use newfound knowledge of causal structures to better predict the effects of interventions (establishment of sufficient training load) to determine optimal training schedules (Pearl, 2010). This suggests the need to consider activity measures from both systems together. All may be performed inside or outside the laboratory; our prototype, Pneumonitor 2 (which can measure changes in thoracic impedance, which is related to changes in the amount of air in the lungs, Młyńczak et al., 2017), appears appropriate for that task, because the results suggested that the depth of breathing is more important than its rate.

This is also indirectly connected with the statement of Fossion et al. (2018), that homeostasis may be quantified using time-series analysis, which might offer several explanations for physiological mechanisms. Use of a portable device allows accounting for various conditions, e.g., body positions. From the analysis, we discovered that the relations between cardiac and respiratory parameters are quite similar for both analyzed body positions. However, the prediction improvement associated with adding a cause parameter was lower for standing than for supine (which is expectable, but can still serve as an additional input). We did not note the effect described by Radovanovic et al. (2018), who reported that even a slight change of body position may change the direction of the cardiorespiratory relationships; however, it merits further study.

Another interesting outcome is the frequency for which the peak G-causality value occurred: about once every 5 s. This is longer than a typical cardiac interval, but could be related to mean breathing rate. This would show a mechanism in which the frequency of breathing serves as a trigger for the causal process even if the depth is more directly related with changes in RR intervals.

Also, as many effects may occur too quickly for the measurement system to track at current time resolution, considering zero-lag elements in the model is in our opinion a crucial step in similar physiological research. This is why (Schiatti et al., 2015) introduced the apparatus for extended Granger causality. Beyond the traditional set of connections, a matrix of instantaneous effects may be prepared. In the analysis, the framework showed the significance of zero-lag effects, not only between durations and amplitudes but also between durations and breathing phases at R peaks (this was not explicitly presented in the text for clarity). The approach seems to also be tied to the kernel regression criteria, which can be exploited for causality inference (Zheng et al., 2012; Vinod, 2016).

Another commonly used measure to assess direction-sensitive connections is the transfer entropy, which can consider equivalent to Granger causality approach for linear Gaussian processes (Schreiber, 2000; Porta and Faes, 2016). The simple implementation of the method based on mutual information distance or generalized correlation sum is written in *TransferEntropy* R package (Mount et al., 2016). Similar to extended Granger causality, the concept was presented earlier by Faes et al. (2013), who introduced so-called compensated transfer entropy, which also includes zero-lags elements into consideration. Instantaneous transfer entropy has also been already used by Valenza et al. (2018) for physiological analyses. Nevertheless, we decided not to incorporate the method in the analysis as there is no output value for transfer entropy such as prediction improvement or *p*-value, that can be easy to interpret for physicians.

### 4.1. Limitations of the Study

The study included only 100 athletes, who formed a heterogeneous group, unevenly distributed in relation to the Mitchell et al. (2005) division. All were studied in the “hot period” 3–4 months before the Olympic Games, which may suggest a state of over-training. Therefore, the findings should be compared with another similar procedure.

As cardiorespiratory parameters and relations (in general) are affected by a number of factors, e.g., age, gender, and levels of physiological or psychological stress, they might have been considered as both confounders and even direct cause variables (Schulz et al., 2015; Widjaja et al., 2015). However, we decided not to include them for clarity and due to the lack of psychologically-oriented questionnaires gathered from athletes. Also, the control group did not answer any questions, so it is not possible to evaluate the differences in their physical preparation relative to the athletes (beyond the fact that no students reported professional participation in sports).

Also, the R peak locations are determined at the 250*Hz* sampling frequency, so the uncertainty in the location of the R peaks is of 4 ms, which may affect the estimates of G-causality computed using such a tachogram.

The collection of only one observation per subject precludes reproducibility analysis. Also, measurements were carried out for a single protocol in an atypical environment. The results of registrations performed outside the laboratory, during normal training, or even with 24 h Holter-based tracking, would yield more condensed and more general findings.

Moreover, the classical Granger causality framework is a linear approach. Several nonlinear generalizations would better fit cardiorespiratory signal specifications. Segments of registrations where analyzed whole, so only a single coefficient was estimated per segment.

Accuracy analysis of the use of causal parameters to distinguish groups was illustrative, not conclusive: there are too little data and the groups are unbalanced. However, one can conclude, those causal parameters may be treated as additional information to standard parameterization, where the accuracies are too weak for them to be used independently.

We did not assume any control variables in the protocol. As this is a retrospective study, we cannot change the respiratory protocol after registration, and also we cannot introduce any interventions to evaluate its effect).

Additionally, our modeling is based on the signals, which cover end-organ responses modulated by multiple levels of complex mechanisms (Dampney, 2015). However, in this study, we attempted a data-driven approach, without including prior knowledge (Młyńczak and Krysztofiak, 2018). Also, one should be aware of the very possible collinearities between X and Y in the original Granger's formulation. This is why we tried to analyze both directions of possible connections and a possible reason for why the causal structure strengths were estimated as mild.

Also, the analysis of two body positions (particularly standing) without segmenting the signals into sub-periods may cause that the results do not cover our mechanisms and processes, and their changes in time. Our choice is, however, dictated by the need to maintain the appropriate signal length for Granger causality approaches.

Finally, we focused only on the data types which can be registered using Pneumonitor 2. However, as the presented protocol was “static,” adding different modalities appears relatively simple. For example, Sobiech et al. (2017) suggested that arterial blood pressure is probably the driver (cause) of both cardiac and respiratory function.

### 4.2. Considerations for Further Studies

The discussion identified several issues for further study:

- how would the accuracy of athletes profiling increase with the addition of causal parameters to standard cardiorespiratory data?
- how would causal links and their strengths differ during natural activity of the subject?
- how coherent are causal parameters for a specific participant in comparable conditions? (reproducibility analysis)
- how would adding restricted breathing to the protocol affect the causal parameters?
- how would the causal links be changed or emphasized with the addition of an arterial blood pressure signal (Silvani et al., 2017; Zhang et al., 2017)?
- can the causal analysis be made more specific and more robust (free or insensitive to collinearity) with model terms conditioned on covariates or even with the addition of a non-linear kernel of Granger-like analysis?
- can the DAG structures be confirmed with a prospective study, which assumes sufficient perturbations and interventions on the cause variable?

One could also evaluate different methods, e.g., based on directional coherence analysis. Schäck et al. (2018) proposed a novel method, robust time-varying generalized partial directed coherence (rTV-gPDC), which carries information about the non-linear connectivity structure using a piecewise linear time-varying moving-average (TVMA) model. It is worth investigating in the presented contexts because the approach assumes a model which is non-linear and which, even more importantly, may adapt over the course of measurement.

## 5. Conclusions

Physiological markers of training performance may be not only biochemical- or cardiac-, but also cardiorespiratory-related. Besides temporal-, spectral-, or information-domain approaches, causal link analysis using pairwise-conditional standard, spectral or extended Granger causality or TiMINo frameworks may introduce new contexts, make the inference more robust and improve result resolution.

We proposed a protocol for elite athletes and controls inspired by the orthostatic maneuver, consisting of free breathing while resting supine and while standing, and took into account various forms of registered data: (1) raw signals sampled at 25*Hz*, (2) raw signals sampled at 2.5*Hz*, and (3) beat-by-beat sequences of cardiac and respiratory parameters.

Based on the data gathered from 100 elite athletes and 20 students included in the control group, we found that temporal causal links are the most evident between the tidal volume signal and the instantaneous heart rate curve (RR intervals or tachogram), and that adding a “cause” variable may improve the prediction of the “effect” variable by 20.3% (median) for supine body positions. While the same causal directions are suggested for standing, the complexity seems higher, as the improvement falls to 14.2% (median). The causal link structures and directions can be parameterized and enable distinguishing athletes from non-athletes with 83.3% accuracy on average. The classification of static and dynamic components can probably be supplemented with causal parameters; however, this requires further investigation and confirmation.

In our opinion, the presented approaches would extend the set of techniques used for profiling training trends by connecting cardiorespiratory data with other psychological information.

## Author Contributions

MM and HK worked on the conceptualization, investigation, project administration, validation, writing and reviewing. MM worked on data curation, methodology, formal analysis, and visualization.

### Conflict of Interest Statement

The authors declare that the research was conducted in the absence of any commercial or financial relationships that could be construed as a potential conflict of interest.

## Supplementary Material

Supplementary Material S1 The Matlab script used for calculating G-causalities.

Supplementary Material S2 The data comprising all calculated G-causalities (columns) for all considered participants (rows); NA - not assigned (when statistically insignificant); stored as an RData file (ready to load into R for analysis).

Supplementary Material S3 The R script consisting of the TiMINo analysis, exploratory data visualization and recursive feature elimination.
